# Roles of quaternary structure and cysteine residues in the activity of human serine racemase

**DOI:** 10.1186/1471-2091-12-63

**Published:** 2011-12-08

**Authors:** Wei Wang, Steven W Barger

**Affiliations:** 1 Department of Geriatrics, University of Arkansas for Medical Sciences, Little Rock AR 72205, USA; 2 Department of Neurobiology & Developmental Sciences, University of Arkansas for Medical Sciences, Little Rock AR 72205, USA; 3 Geriatric Research Education and Clinical Center, Central Arkansas Veterans Healthcare System, Little Rock AR 72205, USA

## Abstract

**Background:**

D-serine is an important coagonist at the NR1 subunit of the NMDA receptor class of glutamate receptors. It is chiefly synthesized in the CNS by serine racemase (SR). Regulation of SR activity is still poorly understood. As step toward developing reagents and methods for investigating SR *in vitro*, we analyzed structure-function relationships of a recombinant enzyme of human sequence.

**Results:**

Michaelis-Menten kinetic analysis indicated a K_M _value of 14 mM and V_max _value of 3.66 μmol·mg^-1^·hr^-1 ^when L-serine was used as a substrate for purified SR. Gel-filtration chromatography and protein cross-linking experiments revealed that dimer is the major oligomeric form of recombinant SR in aqueous solution, though the proportions of monomer, tetramer, and larger aggregates differed somewhat with the specific buffer used. These buffers also altered activity in a manner correlating with the relative abundance of dimer. Activity assays showed that the dimeric gel-filtration fraction held the highest activity. Chemical reduction with DTT increased the activity of SR by elevating V_max_; cystamine, a reagent that blocks sulfhydryl groups, abolished SR activity. Gel-filtration chromatography and western blot analysis indicated that DTT enhanced the recovery of noncovalent SR dimer.

**Conclusions:**

These data suggest that SR is most active as a noncovalent dimer containing one or more free sulfhydryls in the enzyme's active center or a modulatory site. Buffer composition and reduction/oxidation status during preparation can dramatically impact interpretations of SR activity. These findings also highlight the possibility that SR is sensitive to oxidative stress *in vivo*.

## Background

When substantial levels of endogenous D-serine in the mammalian brain were discovered by Hashimoto *et al. *[1], the source of this unusual amino acid became a critical question. This intrigue was settled when Wolosker *et al. *[2] cloned the full-length mouse serine racemase (SR) gene and purified the enzyme from rat brain. These discoveries helped to legitimize D-serine as an endogenous signaling molecule. Colocalization of D-serine with NMDA receptor (NMDAR) expression in most parts of the brain [3,4], together with the fact that degradation of D-serine abolishes NMDAR neurotransmission in different brain regions [5,6], supported the notion that D-serine is an important endogenous ligand for the NMDAR, participating broadly in synaptic events associated with development, plasticity, learning, memory and excitotoxicity. Mice bearing a loss-of-function mutation in SR or targeted deletion of the gene show an 80-90% decrease in brain D-serine levels [7,8]. They also display behavioral phenotypes relevant to schizophrenia, including deficits in spatial object discrimination and long-term memory. These findings are consistent with compelling evidence that NMDAR hypofunction contributes to schizophrenia [9], as well as less complete data implicating D-serine deficiencies in this important human disorder [10]. In addition, evidence indicates that SR is directly involved in age-related deficits in hippocampal cognitive function [11] and the pathogenesis of amyotrophic lateral sclerosis [12].

Highly conserved in mammalian species, SR is a pyridoxal-5'-phosphate (PLP)-dependent enzyme which directly converts L-serine to D-serine. Enzymology studies of SR reveal that SR activity is modulated in many ways by various factors. SR activity is elevated by PLP [2], magnesium, ATP [13,14], calcium [15], and D-serine [16,17]; inhibition of SR activity has been reported for NO [16-18], glycine [19,20], and membrane lipids like PIP2 [21]. Furthermore, SR activity is augmented by physical interactions with other proteins, such as glutamate-receptor-interacting protein (GRIP) [22,23] and protein interacting with C kinase (PICK1) [24,25].

We previously found that proinflammatory conditions in microglia elevated the steady-state levels of an SR dimer that was apparent even in reducing, denaturing gel electrophoresis [26,27]. This phenomenon was accompanied by elevated production of D-serine, suggesting that this stable dimer was responsible for high specific activity. The native structure of recombinant mouse SR was determined by gel-filtration chromatography to exist in a dimer-tetramer equilibrium in solutions [15]. Native SR purified from mouse brain has a relative mobility consistent with a mass of 55 kDa, which is assumed to be a dimer [13]. Recently, the crystal structures of human and rat SR preparations with two mutations (C2D, C6D) also indicated a dimeric structure for this enzyme [28]. It has been reported that oxidation of sulfhydryl groups inhibits SR activity [2,15], but how the redox state of sulfydryl groups affects SR structure is unknown. In this study, the relationship between the structure and activity of SR by reducing and oxidizing sulfydryl group was investigated.

## Results

### Purification and enzymatic characterization of recombinant human SR

For these studies, SR cDNA was amplified by RT-PCR from human RNA and cloned into pTrcHisB, a prokaryotic expression vector that encodes an in-frame polyhistidine domain at the protein's N-terminus. Expressed protein was purified on a nickel-charged affinity resin. Expression of the protein yielded about 4 mg of purified protein from 1 L of bacteria culture. The protein in each fraction collected from affinity columns was detected by SDS-PAGE, which showed a very dense band of SR at 40 kDa (the approximate molecular weight of human SR monomer with the polyhistidine fusion) and indicated that this strategy of affinity chromatography provided protein that was ~90% pure.

**Figure 1:**
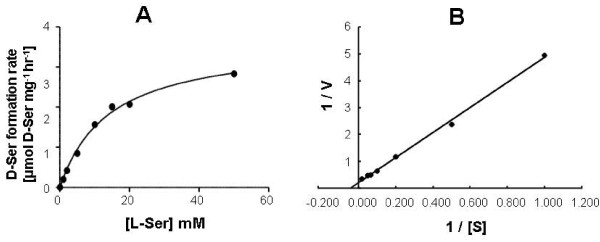
**Kinetic characterization of recombinant human SR**. **A**. Michaelis-Menten plot of the activity of recombinant SR versus increasing concentrations of L-Ser; **B**. Double-reciprocal plot (Lineweaver-Burk) of the data with the axes 1/V versus 1/[L-Ser]. SR activity assay was performed as described under "Methods".

The activity of purified SR was assessed for Michaelis-Menten kinetics (Figure 1), which indicated a K_M _value of 14 mM when L-serine was used as a substrate. The V_max _of SR was 3.66 μmol·mg^-1^·hr^-1 ^at 37°C. These results are similar to data reported previously for recombinant mouse SR protein and purified SR from mouse brain [2,15].

### Oligomeric structure of SR

**Figure 2:**
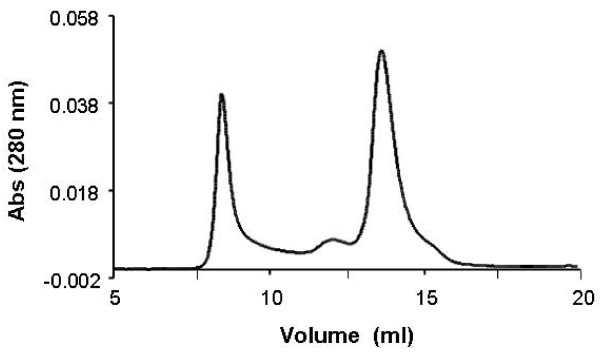
**Gel-filtration chromatography of recombinant SR**. Purified recombinant SR with affinity column was extensively dialyzed against 50 mM phosphate buffer, pH 8.0, 52 mM NaCl. After concentration with Centricon filters, aliquots of 50 μl of ~3 mg/ml of recombinant SR were loaded onto a Superdex HR200 gel-filtration column. Separation was performed at 25°C with flow rate at 0.5 ml/min. The elution buffer was the same as dialysis buffer. Protein detection was performed at 280 nm.

To study the oligomeric structure of SR under native conditions, recombinant SR eluted from affinity column was loaded on a gel-filtration column (Superdex HR200) which resolves proteins in approximately the 10- to 600-kDa molecular-mass range. The elution profile of SR is depicted in Figure 2. Calibration of the column using protein standards and interpolation of the elution volume of SR gave theoretical molecular masses for SR peaks. The first peak eluted at 8.05 min, which fell into the void volume of the column determined by blue dextran, representing a large protein aggregate. The second peak at 11.55 min with theoretical molecular mass 189 kDa probably indicates a tetramer. The third peak at 13.30 min with theoretical molecular mass 87.5 kDa likely represents a dimer. The forth peak appears as a shoulder on the third peak at 14.85 min; its theoretical molecular mass of 45.35 kDa suggests the monomer. The results from gel filtration revealed that dimer is the major oligomeric structure of recombinant SR. There are, however, small amounts of tetrameric and monomeric SR in solution.

**Figure 3:**
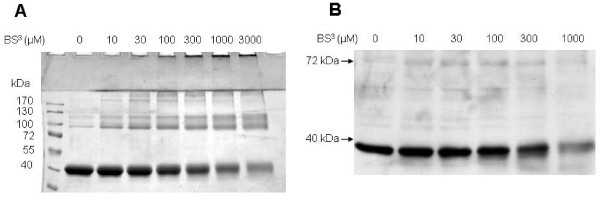
**Stabilization of *in vitro*- and *in vivo*-formed multimers by chemical cross-linker**. **A**. Increasing concentrations of BS^3 ^were incubated with 12.5 μM recombinant SR on ice for 2 h, then the reactions were quenched by addition of Tris-HCl. Reaction samples were analyzed on 10% SDS-PAGE with 2.5% β-mercaptoethanol in the sample buffer. **B**. Increasing concentrations of protein cross-linker BS^3 ^were incubated with lysates from mouse primary astrocytes (0.5 μg protein/μl) on ice for 2 h, and the reactions were quenched by the addition of Tris-HCl. The formation of cross-linked SR protein was analyzed by western blot with an anti-SR antibody.

To confirm the dimeric structure of SR, we fixed protein quaternary structure by using protein cross-linker bis(sulfosuccinimidyl)suberate (BS^3^). BS^3 ^contains an amine-reactive N-hydroxysulfosuccinimide (NHS) ester at each end of an 8-carbon spacer arm, which reacts with protein primary amines at pH 7-9 to form amide bonds resistant to SDS and reducing reagents. First, BS^3 ^was applied to cross-link recombinant SR. The SDS-PAGE (Figure 3A) after the cross-linking reaction showed that with increasing concentrations of BS^3^, SR monomer decreased while dimeric band became denser, indicating that dimer is present in recombinant SR preparations. There was a faint band just above 170 kDa when SR was treated with 10, 30 or 100 μM BS^3^, which may be cross-linked SR tetramer. In addition, one cross-linked band was observed between 100 kDa and 130 kDa; this may be formed by dimeric SR and a small contaminating bacteria protein, based on preliminary sequencing data (unpublished results). In addition to these bands, there were a few high molecular-weight aggregates formed during the cross-linking reaction which were trapped in sample loading wells. To study the quaternary structure of SR in cellular cytosol, BS^3 ^was applied to cytosol extracted from mouse primary astrocytes. After cross-linking reactions, western blot analysis with anti-SR antibody showed that BS^3 ^treatment decreased SR monomer while increasing its dimeric form (Figure 3B), suggesting that SR dimer is also a product of its normal cellular environment.

### Dimer is more active than monomer

**Figure 4:**
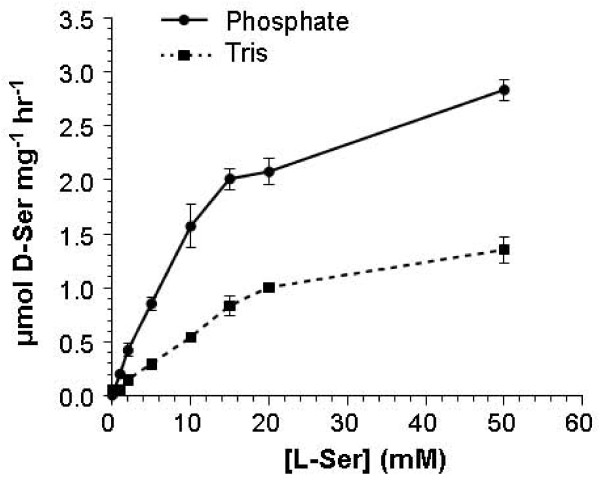
**Influence of buffer components on SR activity**. Recombinant SR purified by affinity chromatography was dialyzed against a phosphate-buffered (solid line) or Tris-buffered solution (dashed line). Then, SR activity of each enzyme preparation was monitored as described under "Methods" except for the reaction buffers, which corresponded to each respective dialysis buffer.

Several different buffers have been used to purify recombinant SR in previously published studies. The effects of Tris-based and phosphate-based buffers on SR activity and structure were tested here. First, SR was purified with affinity chromatography and dialyzed against a phosphate buffer (50 mM sodium phosphate, pH 8.0, 52 mM NaCl) or a Tris buffer (50 mM Tris-HCl, pH 8.0, 150 mM NaCl). SR in each buffer was assessed for activity (Figure 4). Michaelis-Menten analysis revealed a K_M _of 14 mM in phosphate buffer and 24 mM in Tris buffer; the V_max _of the enzyme was 3.66 μmol.mg^-1^.hr^-1 ^in phosphate buffer and 2.06 μmol.mg^-1^.hr^-1 ^in Tris buffer (Table 1). These results indicated that in phosphate buffer SR has higher affinity to L-serine and greater velocity of racemization reaction than in Tris buffer.

**Table 1:** Effect of buffer components on SR activity.

	Phosphate	Tris
K_M _(mM)	14 ± 1.5	24 ± 4.0

V_max _(μmol mg-1 hr-1)	3.664 ± 0.166	2.056 ± 0.180

**Figure 5:**
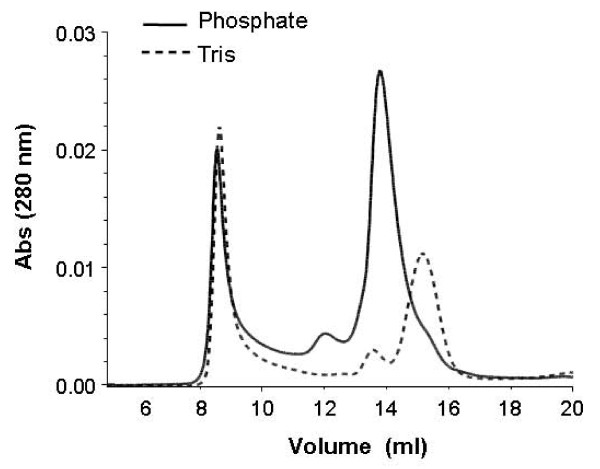
**Influence of buffer components on quaternary structure of recombinant SR**. Recombinant SR purified by affinity chromatography was dialyzed against a phosphate-buffered (solid line) or Tris-buffered solution (dashed line). After concentration with Centricon filters, 50-μl aliquots of ~3 mg/ml of each recombinant SR were loaded onto a Superdex HR200 gel-filtration column and eluted with same buffer as the corresponding dialysis buffer.

To determine what structural change(s) resulted in such a difference in kinetic character of SR in these two buffers, the oligomeric structure of SR was analyzed with gel-filtration chromatography (Figure 5). Interestingly, the elution profiles showed that in phosphate buffer the major conformation of SR was dimer along with a small amount of tetramer, while in Tris buffer the major species was monomer with a small dimer peak. Also, the percentage of large protein aggregates was greater in Tris buffer than in phosphate buffer. To confirm that this difference was not caused by the chromatography itself or other experimental manipulations, 3-(N-morpholino)propanesulfonic acid (MOPS) was used as the buffer in a gel-filtration separation. The results in MOPS buffer were similar to those in phosphate buffer: a large dimer peak and a small tetramer peak (data not shown).

Because the SR monomer peak in phosphate buffer and SR dimer peak in Tris buffer were small, it was impossible to compare dimer and monomer activity in either single buffer. Moreover, isolated monomer or dimer might be expected to re-equilibrate to a proportional mixture of the two after fractionation. But the differing effects of Tris and phosphate buffers on SR quaternary structure provides an opportunity to examine mixtures in which the proportion of dimer differs stably. Gel-filtration fractions of the dimer eluted with phosphate buffer and monomer eluted with Tris buffer were collected and compared in an enzyme activity assay with 10 mM L-Ser. The dimeric preparation produced D-Ser at a rate of 0.253 ± 0.047 μmol.mg^-1 ^.hr^-1^, whereas the value for the monomeric preparation was 0.108 ± 0.010 μmol.mg^-1^.hr^-1 ^(*p *≤ 0.01).

### Cysteine residues are critical to activity and structure of SR

Protein resolving as dimeric in gel-filtration chromatography could potentially include covalent and/or noncovalent dimers. Human SR has eight cysteine residues, creating ample opportunity for a variety of redox-based modifications including disulfide bonds. Such disulfides could act as a bridge to create covalent dimers of SR when occurring intermolecularly, but even intramolecular disulfides could place conformational restraints on the protein's structure. In addition, the free thiol group of a cysteine residue is important for the enzymatic mechanism of many enzymes. We tested the roles of SR's cysteine residues through a coordinated manipulation of redox status and utilization of sizing chromatography.

**Figure 6:**
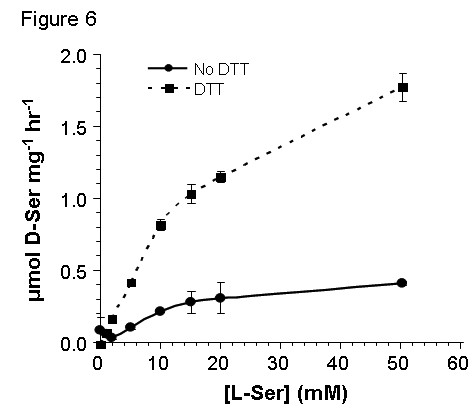
**Chemical reduction elevates SR activity by increasing its V_max_**. SR activity assay was performed in the phosphate buffer with 2.5 μg recombinant enzyme and the indicated concentrations of L-serine; DTT was alternatively excluded (solid line) or included (dashed line) at 0.2 mM. After incubation at 37°C for 30 min, the reaction was terminated by boiling for 5 min. D-ser formation was monitored by a chemiluminescence assay. The data are representative of three different protein preparations.

**Figure 7:**
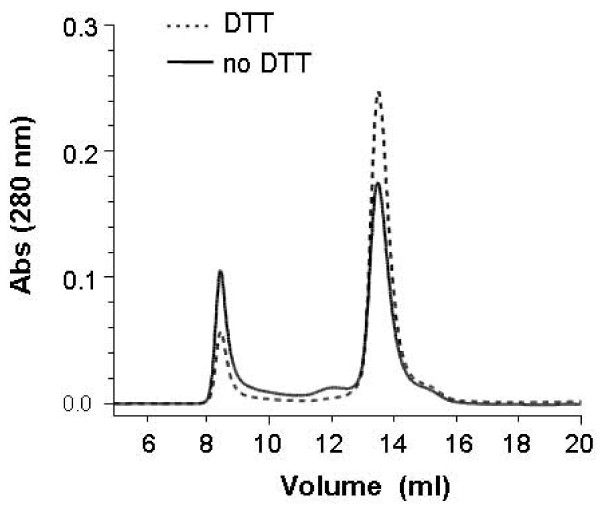
**Effect of chemical reduction on quaternary structure of recombinant SR: native conditions**. Recombinant SR purified by affinity chromatography was dialyzed against the phosphate buffer with (dashed line) or without (solid line) 0.2 mM DTT. After concentration with Centricon filters, 50-μl aliquots of ~10 mg/ml of each recombinant SR were injected onto a Superdex 200 column and eluted with the same buffer as the dialysis buffer.

In preparations of recombinant SR purified only through the initial step of nickel affinity chromatography, reactions containing the thiol-reducing reagent DTT showed higher enzymatic activity; this effect was manifested as a dramatic increase in V_max _(Figure 6). The effect of DTT on SR quaternary structure was assessed with gel-filtration chromatography. The elution profile revealed that DTT decreased the amounts of both the large aggregate and the tetramer while increasing the amount of dimer; monomer amounts were virtually unchanged (Figure 7).

**Figure 8:**
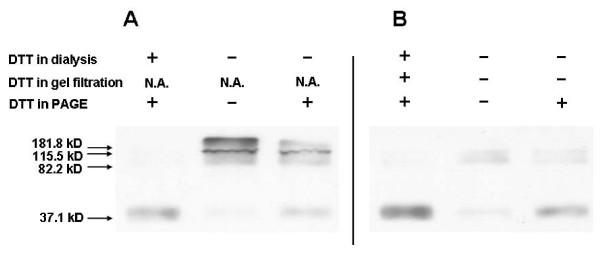
**Effect of chemical reduction on quaternary structure of recombinant SR: denaturing conditions**. **A**. Recombinant SR was dialyzed against phosphate buffers with or without 0.2 mM DTT after elution from the nickel affinity column. **B**. SR preparations in panel A were injected onto a Superdex HR200 column and eluted with same buffer as the corresponding dialysis buffer. Recombinant SR oligomeric species of both protein preparations in A and B were subjected to SDS-PAGE in presence or absence of 0.2 mM DTT in the sample loading buffer. The gels were then immunoblotted with anti-SR antibody.

These results suggest that some of the SR in aggregate and tetramer pools was multimerized by covalent disulfide bonds. This conclusion was supported by western blot analysis of SR eluted directly from the nickel affinity column (Figure 8A) or of dimeric fractions eluted from the gel-filtration column (Figure 8B). For the initial SR preparation obtained from the nickel affinity column, dialysis in the presence of DTT produced protein that entirely denatured to monomer in the presence of SDS. Dialysis in the absence of DTT produced protein that was resolved by non-reducing SDS-PAGE into large aggregates, tetramer, and dimer. These multimers were partially reduced to monomer when DTT was included in the SDS-PAGE sample buffer. Similar results were seen with dimeric SR obtained from the gel-filtration column with or without DTT in the chromatography buffer.

**Figure 9:**
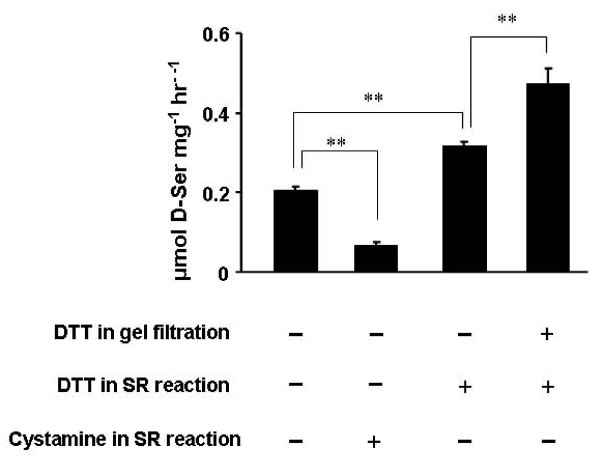
**The role of free sulfhydryls in activity of SR dimers**. Dimeric fractions of recombinant SR were eluted from a Superdex 200 column with or without DTT (0.2 mM) in the mobile phase. SR activity was then assayed. The -DTT fraction was assayed with or without DTT (0.2 mM) or cystamine (1 mM) in the reaction buffer. (**p < 0.0001).

To assess the role of covalent bonds in activity, we compared the activities of SR dimeric fractions eluted from the gel-filtration column with or without DTT added to the chromatography buffer (Figure 9). The activity of SR dimer in the presence of DTT was significantly greater than that of dimer lacking DTT. Addition of DTT into the reaction buffer enhanced the activity of dimer purified without DTT, but this step still produced lower activity than dimer purified in the presence of DTT; this relative ranking generally reflected the level of SR isolated as dimer under native conditions but appearing as monomer in SDS-PAGE (Figure 8B), i.e., non-covalent dimer.

**Figure 10:**
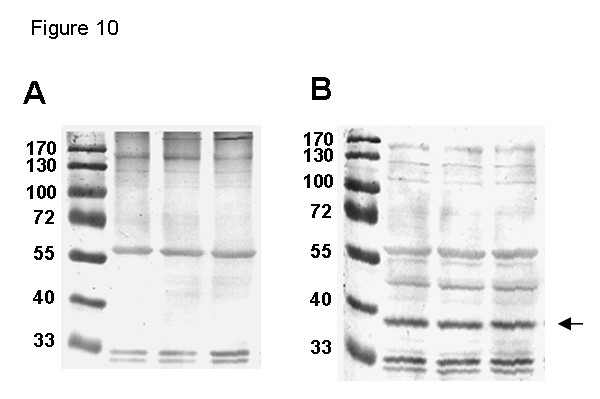
**SR protein from a cellular source oxidizes to dimers and other multimers**. Total cellular protein was prepared from three cultures of mouse primary astrocytes and resolved by 10% SDS-PAGE in the absence (**A**) or presence (**B**) of 2.5% β-mercaptoethanol in the sample buffer. SR protein (arrow) was detected by western blot with anti-SR antibody. Lane 1 contained molecular weight standards (kDa).

To investigate the relevance of disulfide bonds for SR in a cellular context, lysates of mouse primary astrocytes were resolved under non-reducing (Figure 10A) and reducing (Figure 10B) SDS-PAGE. SR protein was detected by western blot analysis. SR monomer was detected only under reducing conditions. Combined with the results of cross-linking experiments (Figure 3B), these data indicate that intermolecular disulfide bonds could be formed in native SR dimers, at least when intracellular redox status is altered, e.g., after depletion of glutathione or aberrant production of reactive oxygen species.

The conformational consequences of disulfides are apparent from the above data, but other types of oxidation of cysteine thiols can also impact their participation in the catalytic mechanism of an enzyme. To further address the role of free sulfhydryl groups in catalysis, we tested the effect of cystamine, a reagent capable of blocking free thiol groups. Cystamine dramatically inhibited the activity of SR dimers (Figure 9).

## Discussion

Physiological activation of the NMDARs requires ligation of both the glutamate binding site (on NR2 subunits) and what has been termed the "glycine_B_" site (on NR1 subunits). There is considerable evidence that the dominant agonist for the NR1 site in most parts of brain is not glycine but D-serine [3,4]. As the only known enzyme to synthesize this novel neurotransmitter in vivo, SR has drawn increasing attention for its role involved in regulation of D-serine synthesis and modulation of glutamatergic synapse activity. In this study we describe the relationship between structural and functional characterization of SR.

Gel-filtration chromatography and cross-linking experiments demonstrated that the major oligomeric structure of recombinant human SR is a dimer. Cross-linking experiments in cell culture also suggested that SR dimer is present in intact cells. One surprising discovery is that recombinant SR had different oligomeric compositions in Tris- and phosphate-based buffers. In phosphate buffer SR is predominantly dimer with a small amount of tetramer, whereas in Tris buffer the major species is monomer with a small dimer peak. By contrast, Smith *et al. *[28] reported that recombinant SR based on the human and rat sequences were dimeric during gel-filtration chromatography with a Tris-based buffer. This distinction may involve differences in the SR primary sequence. Smith *et al. *introduced missense mutations (Cys_2_→ Arg and Cys_8_→ Arg) in order to improve solubility because their production yield was low. Because cysteine residues are so important for SR activity and structure-particularly quaternary structure-those mutants are not comparable to the wild-type sequence we used. In another case, SR was extracted and sequentially purified from mouse brain with an anion-exchange column in Tris-HCl buffer, a Q-sepharose column in Tris-HCl buffer, and hydroxylapatite column in phosphate buffer [13]. The enzyme was then resolved with a gel-filtration column in Tris-HCl buffer, which indicated a species with an apparent size of 55 kDa. Because 55 kDa is between the sizes expected for monomer (37 kDa) and dimer (74 kDa), this finding is difficult to interpret. Though the reason for the buffer's effect is still unknown, this phenomenon permitted a comparison of activity across conditions that favor monomer versus dimer formation. The results support the conclusion that dimer has higher activity as phosphate buffer resulted in both more dimer and more activity. In addition, phosphate buffer is probably closer to physiological conditions than is Tris. Thus, phosphate buffers would seem more appropriate than Tris buffers for biochemical analysis of SR.

The effects of DTT on SR's activity are prominent. The influence of DTT on enzyme's structure includes two possibilities: First, DTT may keep the thiol group of critical cysteine residue(s) in a reduced state. The critical residue(s) may reside in the active site of the enzyme and be important in catalysis of enzyme reactions or reside outside the active site and be critical in modulating the enzyme reactions allosterically. Second, DTT may prevent or break randomly formed disulfide bonds between cysteine residues outside of the active center, thereby helping the protein to fold correctly during synthesis/purification in this recombinant system.

Blocking of free sulfhydryl groups by cystamine dramatically decreased SR activity, which indicates a key role for a cysteine residue(s) in the active site and/or a modulatory site. One previous study [18] mentioned that murine SR protein with Cys_217_→ Ser mutation is basally inactive, which may indicate that Cys_217 _is located in the SR active site and involved in catalysis. Also, Cys_113 _in mouse SR was reported to be S-nitrosylated by NO to form S-nitrosothiol (SNO), leading to a dramatic inhibition of enzyme activity [18]. Structural analysis reveals that the ATP-binding site is in close proximity to Cys_113_, and nitrosylation of Cys_113 _interferes with ATP binding to SR [18]. If the structure of the free thiol group of Cys_113 _is critical for ATP binding to SR, other types of covalent modification of Cys_113 _including a disulfide bond between Cys_113 _and any other cysteine residue is very likely to have the same effect on ATP binding as nitrosylation of Cys_113_. Cys_217 _and Cys_113 _are both conserved evolutionarily, suggesting that disulfide bonds between these residues, either intra- or intermolecularly, accounts for the cross-species similarities documented by Mustafa *et al. *vis-à-vis our results.

In addition to S-nitrosylation, cysteine residue can undergo a broad range of post-translational redox modifications by diverse reactive oxygen species (ROS) and reactive nitrogen species (RNS). The ability to cycle between different redox states and the specificity toward oxidative reactions endow the cysteine residue with properties consistent with it being a participant in redox-based physiological signal transduction or modulation [29]. Hydrogen peroxide can oxidize the cysteine residue in the catalytic center of protein tyrosine phosphatases (PTPs) to sulphenic acid (S-OH), abolishing the catalytic activity of the cysteine residue as a phosphate acceptor [30]. RNS such as NO and peroxynitrite are reportedly able to produce sulphenic acid in the oxidation of thiol compounds [31]. In many cases, sulphenic acid reacts rapidly with other cysteine thiols to form disulfide bonds (S-S). These disulfide bonds can be formed either intramolecularly or intermolecularly, the latter connecting two subunits in one complex or two different quaternary complexes.

As the major covalent connection between peptides, the disulfide bond plays a very important role in protein folding, polymerization, and activity regulation [32], which was also tested in SR here. Under some circumstances, a mixed disulfide bond is formed between cysteine residues in proteins and the small molecule glutathione, an event known as S-glutathionylation (protein-SSG). Accumulating evidence implicates S-glutathionylation in many pathological conditions such as neurodegenerative diseases, cancer, lung diseases, cardiovascular diseases, and diabetes [33]. Under conditions of a robust oxidative burst, sulphenic moieties of cysteine residues can be progressively oxidized to sulphinic acid (R-SO_2_H) and sulphonic acid (R-SO_3_H). While S-nitrosothiol (SNO), sulphenic acid (S-OH), disulfide bonds (S-S), and S-glutathionylation (protein-SSG) can be reduced by glutathione and/or thioredoxin in biological system, formation of sulphinic acid (R-SO_2_H) and sulphonic acid (R-SO_3_H) is believed to be irreversible. Hence, these two hyperoxidized thiol moieties are not considered to be involved in reversible redox signaling [34].

This study also supports the second potential effect of DTT on the enzyme's structure: creating conditions permissive for optimal flexibility of the structure. DTT facilitated release of non-covalent dimer from large aggregates and tetramers, suggesting that during substrate binding and/or catalysis, SR enzyme may need to change its conformation to make the active center more suitable for substrate and the positions of critical amino acids more accurate for catalysis. This is confirmed by recent findings from a SR crystallization study [28], which demonstrated that the tertiary structure of SR has large and small domains connected by a loop region. During the racemization reaction, the small domain appears capable of a large degree of flexibility to correctly orientate the substrate L-serine close to active site for catalysis. Compared to non-covalent dimer, disulfide-linked dimer is less likely to change conformation freely, which may explain its lower catalytic ability. Thus, the flexibility of the enzyme protein appears to be important for its function.

We previously found that inflammatory activation of microglia elevated the levels of a stable SR dimer and D-serine production [26,27,35]. This finding suggested that an apparent covalent dimer was responsible for enzymatic activity. Activation of microglia typically involves a programmatic superoxide production from NADPH oxidase, and this dramatically enhances the oxidation conditions in the cells. It is possible that these conditions result in oxidative crosslinking of the SR subunits. This may involve disulfide bonds, but the species induced by microglial activation was stable to DTT and β-mercaptoethanol. Moreover, the data reported here indicate that disulfide-linked dimers are actually less active than noncovalent dimers, arguing against any connection between formation of the stable dimer and elevated D-serine production in activated microglia. It seems more likely, therefore, that the stable dimer observed in activated microglia results from some chemical conjugation of amino acids other than cystine disulfides. For example, superoxide dismutase has been found to aggregate through several forms of covalent bonds arising from a tryptophanyl radical [36]. It is possible that microglial activation initially results in an elevation of SR expression (via the AP-1 transcription factor, as described [27]), a substantial fraction of which would be active, noncovalent dimer. The subsequent oxidative conditions may then crosslink these dimers via as yet undefined reactions.

## Conclusions

We have obtained data confirming that human serine racemase is most active as a noncovalent dimer. The optimal catalytic activity of the enzyme appears to contain one or more free sulfhydryls; these may be located in the active site or they may effect modulation allosterically. Likely owing to these sulfhydryls, reduction/oxidation status during production and purification of SR can dramatically impact specific activity. We also documented a surprising effect of buffer composition on activity, providing cautionary information for interpretations of SR activity. The structural characteristics of SR obtained from live cells suggest that SR is sensitive to oxidation *in vivo*, perhaps consistent with a scenario in which such modification plays a role in feedback or other forms of regulation.

## Methods

### Recombinant expression of human SR

Full-length SR cDNA was obtained by reverse transcription-polymerase chain reaction (RT-PCR) from human brain mRNA. Two oligonucleotides were used to amplify the cDNA: forward primer, 5'-ATA GGA TCC TGA GCT GAG AAC CAT GTG TG-3' and reverse primer, 5'-GGA AAT GGT GGG AAT TCA GTG GAT CCT AT- 3', where novel BamHI sites (underlined sequences) were introduced before the initiation Met residue and after the "stop" codon. The amplified fragment of DNA (1085 bp) was digested with BamHI and subcloned into pcDNA3.1 plasmid (Invitrogen). The pcDNA3.1-SR plasmid was then digested with BamHI, and the excised fragment of DNA was ligated into the corresponding sites of the pTrcHisB expression vector (Invitrogen). After DNA sequence verification, pTrcHisB-SR vector was transformed into competent BL21 (DE3) *E. coli *(Invitrogen). A 20-ml overnight culture was grown and used to inoculate 480 ml of fresh Luria-Bertani (LB) medium containing 50 μg/ml carbenicillin (Sigma). Typically, the cultures were grown at 37°C with shaking until they reached mid-log (OD_600 _= 0.6). Expression from the pTrcHisB-SR vector was then induced by the addition of 0.75 mM isopropyl-1-thio-β-D-galactopyranoside (IPTG, Fisher Scientific), and the culture was incubated for an additional 3-4 h at 37°C. The cells were collected by centrifugation at 3500 rpm for 30 min and stored at -80°C.

### Purification of recombinant human SR

The frozen cell pellet was resuspended in native binding buffer (50 mM phosphate buffer, pH 8.0, 52 mM NaCl) with 1 mg/ml lysozyme on ice, and the cells were lysed by sonication (six cycles of 30 s with a 30-s cooling period on ice between each cycle). The supernatant of the lysate was obtained by centrifugation at 12,000 × g for 40 min. All the purification steps were performed at 0-4°C. Because the pTrcHisB-SR vector has a hexahistidine tag translated at the N-terminus of SR, purification of the protein was initially performed by affinity chromatography. The cellular supernatant was loaded on to a 5-ml ProBond™ nickel-chelating resin column (Invitrogen). The column was washed with 40 ml wash buffer (50 mM phosphate buffer, pH 8.0, 52 mM NaCl, and 100 mM imidazole). Then the protein was eluted with elution buffer (50 mM phosphate buffer, pH 8.0, 52 mM NaCl, and 250 mM imidazole). The recombinant SR in each fraction was detected by sodium dodecyl sulphate polyacrylamide gel electrophoresis (SDS-PAGE). Fractions with highest concentrations of SR were pooled together and extensively dialyzed against the assay buffer's base [50 mM phosphate buffer, pH 8.0, 52 mM NaCl, 0.2 mM DTT (Fisher Scientific)], first with Tween-20 (0.1%) and then without. After dialysis, proteins were kept at 4°C for structure and activity analysis.

### Enzyme assay

The activity of SR was measured by the velocity of racemization reaction of L-serine to D-serine, the latter of which was assayed by a chemiluminescence assay. The procedure for enzyme reaction was modified and optimized from a described protocol [37]. To minimize the level of the D-serine that contaminates virtually all commercial L-serine reagents, L-serine (Sigma) was catabolized with porcine kidney D-amino acid oxidase (DAO, Calzyme Laboratories) as follows: up to 200 mM L-serine was combined with 1.5 U/ml porcine DAO, 3 U/μl catalase (Sigma) and 84 μg/ml flavin adenine dinucleotide (FAD, Calbiochem) in 0.15 M Tris-HCl, pH 8.3; the mixture was incubated at 37°C overnight, then was heated at 95°C for 10 min to inactivate the enzymes. The supernatant was collected after centrifugation at 14,000 × g for 10 min. Removal of D-serine was confirmed by a D-serine chemiluminescence assay, which indicated that DAO pretreatment reduced the levels of contaminating D-serine by a factor of 20-fold.

SR activity assay was performed with 2.5 μg recombinant enzyme in the presence of 50 mM phosphate buffer, pH 8.0, 52 mM NaCl, 15 μM PLP, 1 mM ATP, 0.2 mM DTT, 1 mM MgCl_2 _and 10 mM pretreated L-serine in a final reaction volume of 50 μl. After the mixture was incubated at 37°C for 30 min, the reaction was terminated by boiling for 5 min. Control incubations for the subtraction of background started with enzyme preparations that had been inactivated by boiling.

The D-serine produced during incubation was monitored by a subsequent chemiluminescence assay. Catabolism of D-serine by DAO generates an α-keto acid, NH_4_^+^, and hydrogen peroxide. The generation of hydrogen peroxide was quantified by the use of peroxidase and luminol, which emits light. A 10-μl aliquot of sample or D-serine standard was added to 50 μl of buffer containing 100 mM Tris-HCl, pH 8.8, 50 mM NaCl, 2 units/ml horseradish peroxidase (HRP, Sigma), 16 μM luminol (Sigma), and 0.8 μg/ml FAD. After a 10- to 20-minute delay, required to decrease a nonspecific luminol luminescence, 60 μl of *R. gracilis *DAO (0.033 U/ml, recombinant; courtesy of L. Pollegioni, U. Insubria, Varese, Italy) was added. A Veritas Microplate Luminometer (Turner Biosystems Inc.) was used to detect luminescence before and after addition of DAO. The value obtained immediately before DAO signal (i.e., after extinction of the nonspecific luminescence) was subtracted from what obtained after DAO, and this remainder was used to calibrate and calculate the D-serine content. K_M _and V_max _of SR reactions were calculated from nonlinear regressions using Graphpad Prism software.

### Gel-filtration analysis of recombinant SR

Aliquots of 50 μl of ~5 mg/ml of recombinant SR were loaded onto a Superdex HR200 gel-filtration column (GE) equilibrated with one of two buffers: 1) 50 mM phosphate buffer, pH 8.0, 52 mM NaCl; or 2) 50 mM Tris-HCl, pH 8.0, 150 mM NaCl. Protein was detected by measuring absorption at 280 nm. The flow rate was kept at 0.5 ml/min. The column void volume was determined with dextran blue. The column was calibrated with proteins from a preparation of gel-filtration standards (Sigma), including cytochrome c (12.4 kDa), carbonic anhydrase (29 kDa), bovine serum albumin (66 kDa), alcohol dehydrogenase (150 kDa), and β-amylase (200 kDa). The calibration curve was obtained by plotting the V_e_/V_o _ratio against the log of the molecular weight, where V_e _is the elution volume of the protein and V_o _is the void volume of the column.

### Protein cross-linking experiments

Recombinant SR (12.5 μM) was incubated with various concentrations of protein cross-linker BS^3 ^(Pierce) in cross-linking buffer [50 mM borate buffer, pH 8.0 with 0.5% Triton X-100 (Sigma), 2 mM EDTA, 150 mM NaCl, and 0.2 mM phenylmethylsulfonyl fluoride (PMSF)] on ice for 2 hours. The reactions were quenched by adding 30 mM Tris-HCl, pH 7.5. The formation of cross-linked SR protein was analyzed with 10% SDS-PAGE. The gels were stained with Coomassie blue. Protein samples from astrocyte cytosol were also cross-linked; lysates were extracted in cross-linking buffer on ice and cleared by centrifugation at 14,000 g for 10 minutes at 4°C. Supernatants (0.5 μg/μl) were incubated with various concentrations of protein cross-linker BS^3 ^in cross-linking buffer on ice for 2 h. The reactions were quenched by adding 30 mM Tris-HCl, pH 7.5. The formation of cross-linked SR protein was analyzed by western blot assay with anti-SR antibody (BD Biosciences).

### Cell culture

Primary mouse glial cultures were established from cerebral cortex of C57BL/6 mice essentially as described [26]. When primary cultures reached confluency (1-2 weeks), flasks were shaken overnight at 200 rpm at 37°C. The supernatant containing microglia was removed and fresh medium was added to the flasks, which were incubated for another 5-7 days. This procedure was repeated three to four times after which cells were trypsinized and resuspended in astrocyte medium comprising minimal essential medium (MEM), 10% fetal bovine serum (FBS), and 0.1 mM L-leucine methyl ester (L-LME, Sigma-Aldrich) to eliminate any remaining microglia. Astrocytes were treated with L-LME for two weeks and were subcultured for experiments by trypsinization.

### Western blot analysis of SR

Amounts of recombinant SR or cell extracts equilibrated for protein content were resolved by 10% SDS-PAGE. Protein was electrophoretically transferred at 100 V for 1.5 h onto nitrocellulose membranes, which were then blocked for 1 h at room temperature with 0.2% I-Block (Applied Biosystems). A monoclonal antibody generated against mouse SR (BD Biosciences) was diluted 1:500 in I-Block and applied overnight at 4°C. The blots were then developed with one of the following two methods: 1) for chemiluminescence detection, the membranes were incubated with alkaline phosphatase-conjugated goat anti-mouse antibody (1:2000) and then developed with the Western-Light detection system (Applied Biosystems); 2) for colorimetric detection, the membranes were incubated with alkaline phosphatase-conjugated rabbit anti-mouse antibody at 1:400, and then developed with BCIP/NBT kit (Vector Laboratories).

### Statistical analysis

Data are expressed as mean ± standard deviation. Data were analyzed using one-way ANOVA followed by *post hoc *comparisons. A *p*-value ≤ 0.05 was considered statistically significant.

## Authors' contributions

WW carried out all the technical aspects of this study, including cloning of human SR, construction of the expression vector, purification, and assay of SR activity. She also drafted the manuscript. SWB initiated the study, participated in its design and coordination, and completed the final revisions of the manuscript. All authors read and approved the final manuscript.

## Authors' information

WW performed these studies in partial satisfaction of her dissertation requirements for a Ph.D. in the Dept. Neurobiol. & Dev. Sci. at Univ. Arkansas Med. Sci.; she is currently a postdoctoral scholar in the Dept. of Geriatrics. She previously obtained a B.S. in biochemistry and an M.S. in molecular immunology from Lanzhou Univ. in Gansu, P.R. China, where she received three academic scholarships. SWB obtained a Ph.D. in cell biology from Vanderbilt Univ. and completed postdoctoral training at the Sanders-Brown Center on Aging at Univ. Kentucky. He is currently a Professor in the Dept. of Geriatrics and in the Dept. of Neurobiol. & Dev. Sci. at Univ. Arkansas Med. Sci.; he is also a Research Health Scientist in the Geriatric Research Education and Clinical Center at the Central Arkansas Veterans Healthcare System. He has served as president of the Arkansas Chapter of the Society for Neuroscience and as a member of the Society for Neuroscience's Committee for Animals in Research; he is currently a member of Council for the American Society for Neurochemistry. He has served on five NIH study sections and special emphasis panels and currently sits on the editorial boards for *Current Alzheimer Research, Journal of Neurochemistry, Journal of Neuroinflammation, Journal of Neuroscience Research*, *Neurobiology of Aging*.
